# Development and Validation of Nomogram Predicting Survival in Resectable Gallbladder Cancer

**DOI:** 10.1002/jhbp.70114

**Published:** 2026-04-07

**Authors:** Shraddha Patkar, Tanvi M. Shah, Mufaddal Kazi, Sadhana Kannan, Gurudutt Varty, Vikas Ostwal, Anant Ramaswamy, Prabhat Bhargav, Prajakta Barve, Mahesh Goel

**Affiliations:** ^1^ Department of Surgical Oncology Tata Memorial Hospital and Homi Bhabha National Institution Mumbai India; ^2^ Department of Biostatistics Advanced Centre for Treatment, Research and Education (ACTREC) Navi Mumbai India; ^3^ Department of Medical Oncology Tata Memorial Hospital Mumbai India

**Keywords:** adjuvant treatment selection, gallbladder cancer survival, predictive nomogram

## Abstract

**Background:**

Gallbladder cancers (GBCs) are aggressive tumors with limited improvement in outcomes over decades. Accurate prediction of survival is crucial for tailored treatment and improved outcomes.

**Objective:**

To develop a nomogram predicting overall survival (OS) in resectable GBCs.

**Methods:**

A retrospective analysis of 1045 patients of resected gallbladder cancer between January 2010 to December 2022 was done. A predictive nomogram was built and was internally validated.

**Results:**

The nomogram included age, original T stage, original N stage, and lympho‐vascular/perineural invasion, CA 19.9, incidental versus non‐incidental GBC, and grade. The Harrell's concordance index for the nomogram was 0.677. The area under the ROC curves for 1‐, 3‐, and 5‐year OS were 72.12, 72.64, and 71.15 respectively. Patients were stratified into low‐risk and high‐risk groups based on a threshold score of 22.48, with significantly different median OS (not reached vs. 31.4 months, *p* < 0.001).

**Conclusions:**

The developed nomogram demonstrates good discriminative capacity and risk stratification in patients undergoing surgery for GBC and may be utilized for better selection and application of adjuvant treatment strategies, potentially improving outcomes.

## Graphical abstract

Patkar and colleagues developed and internally validated a nomogram predicting overall survival after resection of gallbladder cancer using a large cohort of 1045 patients. The model demonstrated good accuracy and discriminative capacity, and stratified patients into prognostically distinct risk groups, supporting more objective selection and application of adjuvant treatment strategies.

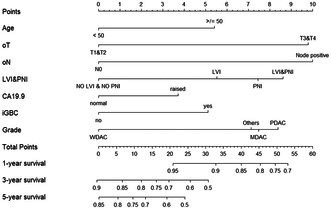

## 1 Introduction

Gallbladder cancers (GBCs) are aggressive tumors with very little improvement realized in the outcomes over decades. Traditionally, the reported 5‐year overall survival (OS) after curative resections is only 20%–30% [1] and has largely remained dismal over decades [2]. Adjuvant and neoadjuvant therapies have been shown to improve outcomes; with 3‐year OS reported in one large series to be 94.1%, 82.6%, and 48.2% for Stage I, II, and III respectively [3]. The published evidence is heterogenous in terms of indications and protocols, and often GBCs are included within the broad spectrum of biliary tract cancers (BTCs) [4, 5, 6]. As GBCs represent a very distinct clinical entity differing from other sub‐sites of BTCs in terms of chemosensitivity and radiosensitivity and the surgical approaches, it is imperative to develop tools which can accurately predict the survival and risk of recurrence in an individual patient, thereby guiding precise and tailored treatment, with an aim to improve long‐term outcomes. The present study aims to develop and validate a nomogram predicting OS in patients with GBCs undergoing curative treatment, which can be used as objective tools for supplementing clinical decision making.

## 2 Methods

The study follows a retrospective observational cohort design. A prospectively maintained database of patients operated for GBCs at a tertiary care cancer center from January 2010 till December 2022 was collated to develop the nomogram. Both per‐primum and incidental GBCs (iGBC) were included. Patients found to have unresectable disease at exploratory laparotomy were excluded.

The data collected included the following preoperative variables: age, gender, carbohydrate antigen 19–9 (CA 19.9) levels, presence of obstructive jaundice (OJ), and original (radiological) T stage (oT) at presentation (before neoadjuvant chemotherapy [NACT]). This includes radiological T stage for non‐incidental tumors and for incidental GBC with residual/recurrent disease. For incidental gallbladder cancers without residual disease, the pathological T stage (pT) of the index surgery was considered as original T stage. Similarly, the original (radiological) N stage at presentation (oN) was included. Treatment‐related variables included neoadjuvant treatment and type of surgery (radical/revision cholecystectomy or extended resections). oT and oN stage were included to negate the confounding effect of pathological downstaging following NACT and therefore maintain the generalizability of the model. Similarly, NACT was not included as a factor in the analysis, since it is more of a predictive factor (than a prognostic factor) and may not be considered a standard of care uniformly, thereby limiting the generalizability of the model. Histopathological variables included pathological T stage (pT), pathological N stage (pN), tumor grade margin negative (R0) versus positive (R1 and R2) resection, lympho‐vascular invasion (LVI), perineural invasion (PNI), and lymph node ratio (LNR; defined as the ratio of positive lymph nodes to the total number of nodes dissected). Staging was as per the 8th edition of TNM staging by the American Joint Committee on Cancer (AJCC) and International Union Against Cancer (UICC). Postoperative complications were defined using the Clavien–Dindo classification system, and Grade 3 or above were considered significant and recorded [7]. Adjuvant treatment details were also recorded. Data collection was in accordance with the Declaration of Helsinki [8]. The study has been approved by the institutional review board (Project number: 4816/TMH). OS was calculated from the date of diagnosis until last follow‐up or death. For iGBC, the OS was calculated from the date of the index cholecystectomy until last follow‐up or death. Patients lost to follow‐up were censored at the last date of the last outpatient visit.

All patients underwent evaluation and treatment as per the standard institutional protocol after discussion in a multidisciplinary tumor board. The treatment protocols have been published alongside previous report from the institution [3]. Patients with early disease at presentation (defined as clinico‐radiologically, stage T1‐T2N0) underwent upfront radical cholecystectomy (en‐bloc resection of gallbladder with liver wedge and periportal lymphadenectomy). Patients with locally advanced gallbladder cancers (LAGBC) defined as per the “TMH‐Criteria” (cT3‐T4, node positive, vascular abutment, iGBC with residual disease/node positive), were given 4 cycles of NACT according to the institutional protocol [9]. The chemotherapy regimen commonly used was gemcitabine: 1000 mg/sq‐m and cisplatin: 25 mg/sq‐m in most cases. Patients with iGBC were treated on similar lines [10]. Patients with good or partial response and/or stable disease after NACT, underwent radical cholecystectomy. Our standard technique for radical cholecystectomy includes en‐bloc resection of gallbladder with a 3‐cm wedge of liver all around the tumor to achieve a margin negative resection, and a complete periportal lymphadenectomy (including stations 8a, 8p, 12a, 12b, 12c, and station 13). Similarly, a revision surgery (for iGBC) includes a 3‐cm wedge of liver all around the gallbladder fossa (since gallbladder is not in situ), with a complete periportal lymphadenectomy. We do not routinely perform an anatomical hemi‐hepatectomy. The cystic duct margin is sent for frozen section examination and if reported positive, we proceed with an extrahepatic bile‐duct excision. If an additional organ; other than liver segment and bile duct (e.g., stomach/duodenum/colon was infiltrated) requiring en‐bloc excision, the procedure was classified a radical cholecystectomy with additional organ resection. The final histopathology reporting includes details of tumor size, differentiation, parenchymal and cystic duct margin status, presence of LVI and PNI and presence of any discontiguous tumor nodules/deposits in the resected liver parenchyma. Adjuvant chemotherapy was given to patients (pT1b onwards or any node positive disease) who recovered well from surgery. As an institutional protocol, patients with pT1b disease with other high‐risk features like LVI/PNI/poor differentiation and pT2N0 patients received single agent Capecitabine/S1 regimen as adjuvant chemotherapy. Most other patients received a gemcitabine–cisplatin doublet regimen. Few patients with multiple high‐risk factors (pT4/N2) received gemcitabine–cisplatin–nab‐paclitaxel triplet regimen. Patients were followed every 3 month for first 2 years and then 6 month thereafter till 5th year of follow‐up.

### 2.1 Statistical Analysis

Statistical analysis was performed using Statistical Product and Service Solutions (SPSS) version 26 (IBM Corporation, Armonk, NY, USA) and the R software, Version 2024; 12.1 + 563. Continuous variables were described as median (interquartile range [IQR]) while categorical variables were described as number (%). Differences in baseline characteristics were assessed using the Kruskal–Wallis test for continuous variables and chi square or Fisher's exact tests for categorical variables. The age group, gender, T stage, N stage, grade, LVI, PNI, OJ, NACT, and adjuvant chemotherapy were all included as categorical variable, while L/N ratio was used a continuous variable. Survival curves for OS were obtained using the Kaplan–Meier method. Follow‐up data was missing for 54 (5%) patients. These patients were censored at the date of last follow‐up for survival estimation.

Prognostic factors affecting survival, were initially analyzed using univariate Cox regression. Variables with a *p*‐value < 0.05 on the univariate analyses were then entered into a stepwise multivariate Cox regression analysis. The best multivariate Cox regression model outputs by stepwise regression was then utilized to construct a predictive nomogram for OS, based on the Akaike information criterion (AIC) for goodness of fit.

The discriminative power of the nomograms for 1‐, 3‐, and 5‐year OS and DFS was evaluated using the time‐dependent Harrell's concordance index (C‐index). The predictive accuracy of the nomograms for 1‐, 3‐, and 5‐year OS and DFS rates was assessed using calibration curves and receiver–operator curves (ROC).

The risk stratification functions of the nomograms were based on the individualized risk scores, and the optimal threshold for determining the high‐risk and low‐risk groups was chosen according to the maximum Youden's index. Survival outcomes were compared between the risk groups using Kaplan–Meier curves and log‐rank tests. The reliability and generalizability of the nomograms were further assessed by internal validation using 1000‐times boot strap resampling, using the same approaches, including time‐dependent C‐index, calibration curves, ROC curves, and risk‐stratified Kaplan Meier curves. A web‐based calculator was made based on the nomogram using the rms and shiny packages in R.

## 3 Results

Over the given period, 1056 patients underwent surgery for GBC. Patients with less than 6 months follow‐up and missing data were included from the analysis. The nomogram was developed using data from 1045 patients with resected GBC, which included 576 (55.2%) of iGBC. The baseline demographic and treatment characteristics of the cohort are summarized in Table 1. The median age of presentation was 52 years, 673 (64.4%) patients had T1–T2 disease while 372 (35.6%) had T3–T4 disease. Two hundred and fifty‐four (24.5%) patients had node positive disease. Two hundred and fifty‐eight (24.6%) patients had raised CA 19.9 level (> 37 IU/mL) with the median level in these patients being 150 IU/mL (range: 38–373 957 IU/mL). Two hundred and eighty‐four (27.2%) patients received NACT and 289 (97.3%) completed all planned cycles of NACT. Complete (R0) resection was achieved in 1025 (97%) patients and the median lymph node yield was 10 (range: 4–41); with 891 (84.4%) patients having 6 or more nodes sampled. Seven hundred and forty‐one (71%) patients received adjuvant chemotherapy; including 291 (38.9%) patients receiving capecitabine or S1 monotherapy, 375 (50.1%) receiving Gem‐based doublet therapy and 38 (5.1%) patients received triplet (gemcitabine–cisplatin–nab‐paclitaxel) regimen. Seven hundred and sixteen (95.7%) patients completed all planned cycles of adjuvant chemotherapy. The types of surgeries performed are summarized in Table 1. Thirty‐nine (3.7%) of these were robotic surgeries.

**TABLE 1:** Baseline characteristics of the cohort.

Baseline character	Category of variable	Frequency
Median age (years)		52
Gender	Male	306
	Female	739
Obstructive jaundice	Yes	82
	No	963
oT	T1	157
	T2	516
	T3	362
	T4	10
oN	Node negative	789
	Node positive	256
CA 19.9	Range	2–373 957
	Proportion of patients with raised CA 19.9 (> 37 IU/mL)	258 (24.6%)
	Median levels in patients with raised level (> 37 IU/mL) CA	150 IU/mL
Surgeries	Radical cholecystectomy	355 (34.0%)
	Revision surgery	576 (55.2%)
	Radical cholecystectomy with bile‐duct excision	74 (7.0%)
	Radical cholecystectomy with additional organ resection	40 (3.8%)
pT	T1	154
	T2	471
	T3	411
	T4	9
pN	N0	789
	N1	227
	N2	29
Median nodal yield		10
Cystic duct margin	Negative	1018
	Positive	27
Completeness of resection	R0	1025
	R1	24
	R2	6
Grade	WDAC	103
	MDAC	636
	PDAC	229
	Others	77
NACT	Yes	284
	No	761
LVI and PNI	No LVI/PNI	785
	LVI	95
	PNI	85
	LVI and PNI	80
Adjuvant	None	304
	CT	699
	CTRT	42

Abbreviations: CT, chemotherapy; CTRT, chemoradiation; LVI, lympho‐vascular invasion; MDAC, moderately differentiated adenocarcinoma; NACT, neoadjuvant chemotherapy; oN, original (radiological) N stage; oT, original (radiological) T stage (before initiation of neoadjuvant treatment); PDAC, poorly differentiated adenocarcinoma; pN, final (pathological) N stage; PNI, perineural invasion; pT, final (pathological) T stage; R0, microscopic negative resection margin; R1, microscopic positive resection margin; R2, macroscopic positive resection margin; WDAC, well‐differentiated adenocarcinoma.

### 3.1 Overall Survival (OS) and Factors Affecting OS

The median follow‐up of the cohort, using the reverse Kaplan–Meir method was 64.8 months (95% CI: 60.3–69.4 months); there were 454 deaths and 491 recurrences. The median OS was 66.5 months. On multivariate analysis, age (HR: 1.406 [1.157–1.709]; *p* = 0.001), iGBC (HR: 1.377 [1.12,1.694]; *p* = 0.002), oT stage (HR: 1.904 [1.543,2.35]; *p* < 0.0001), oN stage (HR: 2.475[2.042,3]; *p* < 0.0001), LVI and PNI (HR: 2.279 [1.684,3.084]; *p* = 0.021), and grade were significant factors predicting OS (Table 2).

**TABLE 2:** Univariate and multivariate analysis for factors affecting overall survival (OS).

Variable	Category	Total numbers	Events	Univariate HR (95% CI)	*p*	Multivariate HR (95% CI)	*p*
Age	< 50 years	428	168	Reference		Reference	
	≥ 50 years	617	286	1.33 (1.099,1.609)	0.003	1.406 (1.157,1.709)	0.001
OJ	No	963	414	Reference			
	Yes	82	40	1.377 (0.995,1.906)	0.054	0.960 (0.675,1.366)	0.822
iGBC	No	469	206	Reference			
	Yes	576	248	0.936 (0.778,1.127)	0.048	1.377 (1.12,1.694)	0.002
oT Stage	T1&T2	673	229	Reference		Reference	
	T3&T4	372	225	2.272 (1.889,2.732)	< 0.001	1.904 (1.543,2.35)	< 0.001
oN Stage	Node negative	789	290	Reference		Reference	
	Node positive	256	164	2.475 (2.042,3)	< 0.001	1.76 (1.337,2.317)	< 0.001
R status	R0	1015	434	Reference		Reference	
	R1	24	14	1.981 (1.162,3.376)	0.012	1.388 (0.732,2.632)	0.316
	R2	6	6	9.592 (4.261,21.592)	< 0.001	7.333 (2.768,19.425)	< 0.001
Grade	WDAC	103	25	Reference		Reference	
	MDAC	636	273	2.047 (1.359,3.084)	0.001	1.55 (1.022,2.351)	0.039
	PDAC	229	118	2.83 (1.838,4.359)	< 0.001	1.627 (1.037,2.552)	0.034
	Others	77	38	2.031 (1.226,3.364)	0.006	1.632 (0.97,2.745)	0.065
LVI‐PNI	No	785	303	Reference		Reference	
	LVI	95	52	1.539 (1.147,2.066)	0.004	1.30 (0.962,1.757)	0.087
	PNI	85	50	1.889 (1.4,2.548)	< 0.001	1.482 (1.085,2.024)	0.013
	LVI&PNI	80	49	2.279 (1.684,3.084)	< 0.001	1.425 (1.032,1.969)	0.032
LN* Ratio				5.506 (3.55,8.54)	< 0.001	1.602 (0.768,3.343)	0.209
Adjuvant Treatment	Nil	304	98	Reference			
	CTRT	42	25	2.639 (1.698,4.099)	< 0.001	0.93 (0.525,1.646)	0.803
	CT	699	331	1.789 (1.427,2.243)	< 0.001	1.208 (0.948,1.540)	0.126
CA19.9	Normal	787	315				
	Raised	258	139	1.598 (1.308,1.951)	< 0.001	1.219 (0.972,1.528)	0.056

Abbreviations: CT, chemotherapy; CTRT, chemoradiation; LN ratio, lymph node ratio; LVI, lympho‐vascular invasion; MDAC, moderately differentiated adenocarcinoma; OJ, obstructive jaundice; oN, final (pathological) T stage; oT, initial (clinico‐radiological) T stage (before initiation of neoadjuvant therapy); PDAC, poorly differentiated adenocarcinoma; PNI, perineural invasion; R0, microscopic margin negative resection; R1, microscopic margin positive resection; R2, gross margin positive resection; WDAC, well‐differentiated adenocarcinoma.

* Lymph node ratio (LNR) was entered as a continuous variable ranging from 0 to 1. Therefore, the reported HR of 1.24 technically reflects the effect of a one‐unit increase in LNR—that is, comparing LNR = 0 with LNR = 1—corresponding to a 24% higher hazard of death. However, we acknowledge that this comparison represents the extreme ends of the scale and is not clinically intuitive. To provide a more meaningful interpretation, we considered smaller increments. When expressed per 10% (0.1 unit) increase in LNR, the corresponding hazard ratio is 1.24^0.1 ≈1.02. This indicates that for every 10% increase in lymph node ratio, the hazard of death increases by approximately 2%.

### 3.2 Construction and Validation of Nomogram Predicting OS

**FIGURE 1:**
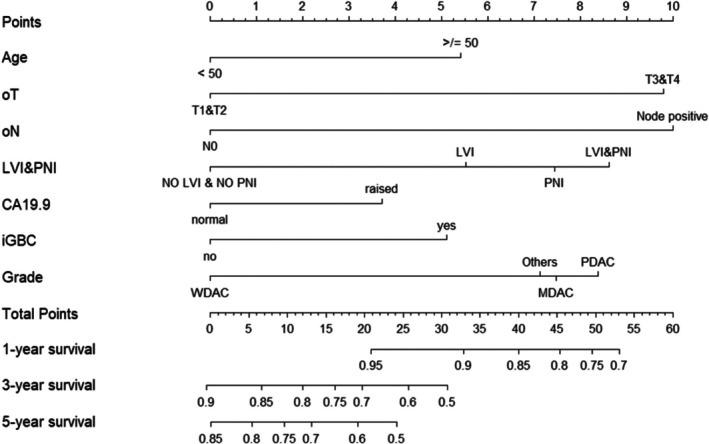
Nomogram predicting overall survival (OS).

The model for OS was developed based on AIC for goodness of fit. The nomogram included seven factors namely, age, oT stage, oN stage, LVI‐PNI, CA 19.9 (raised/normal), iGBC (yes/no) and grade. Age of < 50 was assigned a score of 0 while 50 or more was assigned a score of 5.2. oT stage of T1/T2 was assigned a score of 0 while the score for T3/T4 was 9.3. oN negative disease had a score of 0 while oN positive disease was scored 10. The scores for LVI and PNI were stratified as 0, 5.2, 7.2, and 8.4 for no LVI‐PNI, LVI positive but PNI negative, PNI positive but LVI negative and both positive respectively. Normal CA19.9 was assigned a score of 0 while raised CA 19.9 was given a score of 3.4. Non‐incidental GBC received a score of 0, while iGBC was scored as 5.2. Lastly, grades were stratified as WDAC, MDAC, PDAC, and others and scored as 0, 7.2, 8.2, and 7 respectively (Figure 1).

For example, a patient of age 52 with non‐incidental GBC, with oT stage of T2, node negative disease with LVI and PNI positivity, raised CA19.9 and MDAC histology will have a total score of 24.2, corresponding to 1‐year, 3‐year, and 5‐year survival probabilities of > 90%, 60%, and 47% respectively.

**FIGURE 2:**
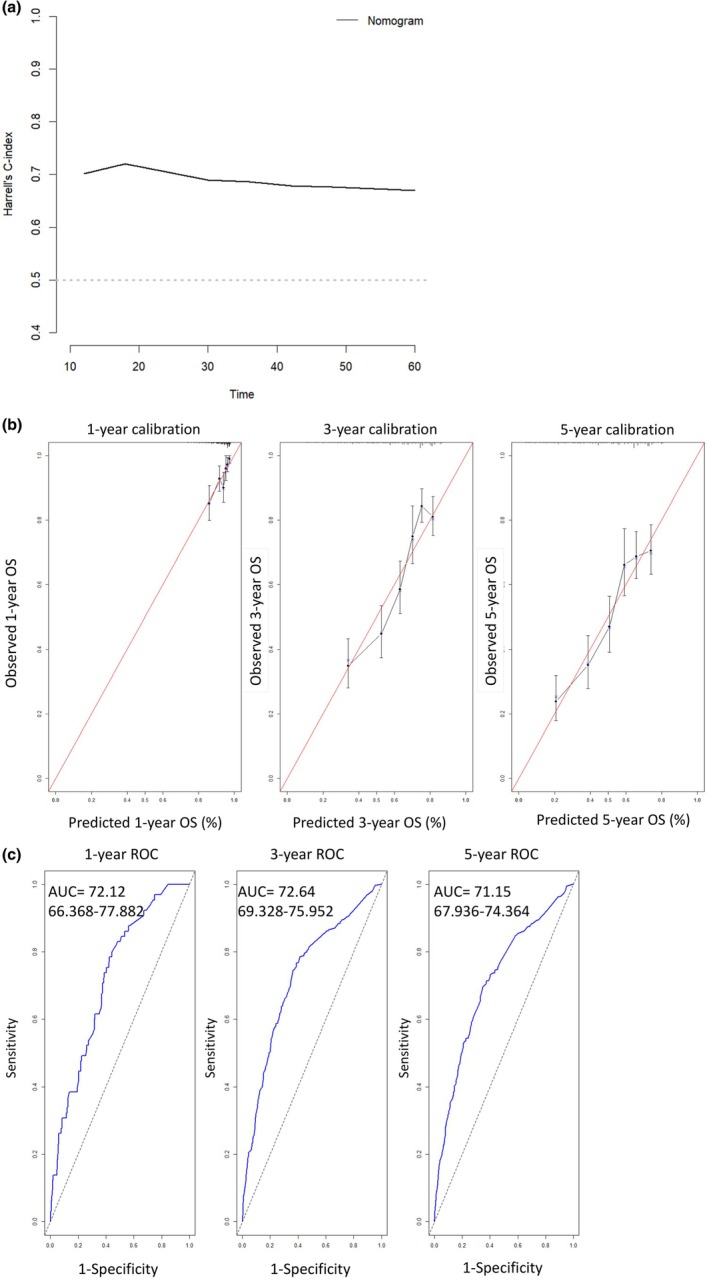
(a) Discriminative power of the nomogram (time‐dependent Harell's C‐index). (b) Calibration curves for accuracy in predicting 1‐year, 3‐year, and 5‐year overall survival (OS). (c) Receiver–operator (ROC) curves for predictive accuracy for 1‐year, 3‐year, and 5‐year OS.

The discriminative power of the model was assessed by calculating the time‐dependent Harrell's concordance index (C‐index), which was 0.684. Bootstrap validation was applied to estimate the overfit and the adjusted C‐index after 1000 iterations was 0.677, demonstrating a good discriminative power (Figure 2a). The predictive accuracy of the nomogram for 1‐, 3‐, and 5‐year OS was assessed using calibration curves (Figure 2b) and ROC curves. The calibration curve slope was 0.939 showing minimal overfitting. The ROC curves consistently yielded AUC of 72.12, 72.64, and 71.15 for 1‐, 3‐, and 5‐years OS (Figure 2c).

**FIGURE 3:**
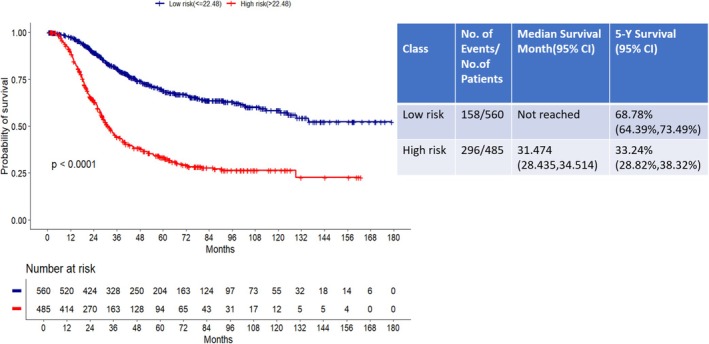
Risk‐stratified overall survival.

The risk stratification function threshold was determined by the Youden's index obtained from the ROC curves. The score corresponding to the Youden's index was 22.48. Patients with a score of ≤ 22.48 were stratified as low‐risk, while those with > 22.48 were stratified as high‐risk. The median OS was significantly different in the two groups: low‐risk group—not reached and high‐risk group 21.4 months (28.4–34.5 months); (*p* < 0.001). The 5‐year OS of the two groups was 68.7% and 33.2% respectively (Figure 3).

The American Joint Committee on Cancer (AJCC)‐ Tumor, Node, Metastasis (TNM) staging system was found to have a C‐index of 0.67, suggesting a good discriminative power; however, the AUC for 1‐year, 3‐year, and 5‐year OS were 68.3 (61.97–74.71), 70.75 (67.28–74,22), and 66.43 (62.55–70.31) respectively suggesting a fair‐to‐low accuracy.

A web‐based online calculator predicting 1‐year, 3‐year, and 5‐year survival probabilities has been devised to aid in prognostication for individual patients.

https://tmhstats.shinyapps.io/PREDICT/.

## 4 Discussion

Nomograms are being increasingly used as effective tools for prognostication in various cancer types [11]. Nomograms can prove useful in various clinical settings—preoperative patient selection, postoperative planning of adjuvant treatment, and planning treatment of advanced cancers. Unlike the more generalized TNM staging, these nomograms can provide precise prognostic information for an individual patient for better clinical decision making.

The existing AJCC 8th edition staging system for GBCs is comprehensive and prognosticates stage‐wise well [12]. However, it does not take into account factors like grade of tumor, LVI and PNI, and margins of resection into consideration. While T and N stages are the most important prognostic factors, tumor grade [13], presence of LVI [14] and PNI [15], completeness of resection (R0/R1/R2) [16] and lymph node ratio [17] and CA 19.9 have shown independent prognostic significance. The multivariate analysis in our study has also shown similar consistent results. The models were built with the aforementioned factors, and the best‐fit model was chosen and thereupon validated. The model has a very good predictive accuracy and discrimination power (C‐index = 0.677), while most studies have found the AJCC‐TNM staging to have a moderate discrimination power (C‐index = 0.59–0.63) [18, 19, 20].

Although the C‐index using AJCC‐TNM staging for our cohort was 0.67, suggesting a good discriminative power, the accuracy of the AJCC‐TNM staging was fair‐to‐low. Moreover, the nomogram is a more objective tool for decision making. There has been conflicting evidence about the survival benefit of adjuvant therapy in the landmark trials. The ASCOT trial (14% patients with GBCs) has shown OS benefit (3‐year OS: 77.1% vs. 67.7%) with single agent S1 [6] and the BILCAP trial (18% patients with GBCs) has shown OS benefit in the per‐protocol (but not intention‐to‐treat) analysis with single agent Capecitabine (49.6 vs. 36.1 months) [4]. Whereas, the PRODIGE‐12 (20% patients with GBCs) failed to show any benefit in terms of recurrence free survival [5]. Owing to the lack of uniformity of evidence, the utilization of adjuvant chemotherapy for GBCs has been variable with substantial underutilization rates [21]. The nomogram is more likely to come handy in such clinical situations. For example, while there is evidence on the role of adjuvant therapy for high‐risk gallbladder cancers [22], there is no consensus on the definition of high‐risk disease. Our institution has formulated and used the “TMH‐Criteria” for risk stratification of GBCs for the purpose of triaging patients to NACT or surgery [9]. But there is lack of a similar standardized system in the adjuvant setting. The nomogram in our study risk stratifies patients as high‐risk or low‐risk of cancer related death or recurrence. This may provide ground for superior selection of patients toward appropriate adjuvant treatment. For example, a patient with T2N0 disease may be considered as low risk (based on the AJCC‐TNM staging) and in absence of a standard policy may be considered for observation or chemotherapy based on individual institutional protocols. Considering two different clinical scenarios; a 45‐year‐old patient with T2N0 disease without LVI and PNI, normal CA 19.9 and MDAC, will have a nomogram‐predicted, 5‐year survival probability of 77.59%; on the other hand, for a 55‐year‐old patient with T2N0 disease with LVI and PNI positive PDAC and raised CA 19.9 the 5‐year survival probability drops down to 45.88%, in which case a better‐informed decision can be taken and the patient could best be considered for adjuvant chemotherapy.

The best fit adjuvant treatment regimen also continues to evolve both in terms of number of chemo‐therapeutic agents (single agent vs. doublet vs. triple‐drug regimen) and the disease specific sensitivity [23]. The nomogram provides a good opportunity to select patients as per risk stratification to varying chemotherapy regimens in clinical practice and future trials.

Some nomograms have been developed previously for GBCs; however, their clinical application is limited, mostly because of the smaller sample size of the cohorts [2, 24, 25]. Lohman et al. developed a prediction model based on a Dutch nationwide cohort (*n* = 380) and validated it using an Australian cohort (*n* = 66) [2]. Wu et al. developed a Bayesian network model and a nomogram model based on data of 698 patients across six hospitals in China [24] however, both models were not externally validated. Another Chinese study by Deng et al. (*n* = 287) included some novel factors into the development of the nomogram, such as body mass index, the Charlston comorbidity index, platelet count, serum albumin, CA‐125, among others, and validated it on a small cohort of (*n* = 85) patients [25]. A few large series based on Surveillance, epidemiology, and end results (SEER) database have included treatment related factors in the nomogram development which are heterogenous within the training cohort and hence not generalizable [26, 27, 28].

The nomogram in the current study has been developed on a large cohort of patients (*n* = 1045), all of whom have been treated using standard surgical principles and perioperative management. This essentially eliminates the confounding effect of treatment‐related factors and provides an opportunity to capture the true prognostic factors associated with the disease biology. This in turn makes the nomogram generalizable. The data of the study comes from a geographical zone harboring endemic proportions of disease.

The study has a few limitations. Firstly, the nomogram is not externally validated. Secondly, the nomogram is based on postoperative variables like LVI and PNI and hence can be used for decision making in the adjuvant setting only. There is still an unmet need to develop models which can predict prognosis based on preoperative variables. This may facilitate better identification of high‐risk patients who may benefit from neoadjuvant therapies and in some cases, may even prevent futile surgical exercise. The “TMH‐criteria”, developed in 2018 was one of the 1st objective criteria to define the role of NACT [9]. Similarly, the recent multicentric Phase II trial: KHBO1201 [29], utilized PET positive lymph nodes as a criterion for selecting patients for NACT. However, a robust model including these clinico‐radiological characteristics needs to be developed to objectively define the role of NACT.

Recent collaborative studies have highlighted the futility of aggressive treatment of locally advanced and advanced tumors [30]. Studies have also identified a high risk group likely to fail early with a futile surgical exercise [31]. While we await the development of precision oncology to improve the prognosis of GBC, it is imperative to refine patient selection towards appropriate available treatment options. The current nomogram is an effort in the same direction. Future research is needed to develop similar nomograms in the pretreatment settings.

## 5 Conclusion

The nomogram developed in the study has shown good discriminative capacity and potential for risk stratification of patients undergoing surgery for GBC. This may be utilized in clinical practice for better selection and application of adjuvant treatment strategy.

## Funding

The authors have nothing to report.

## Conflicts of Interest

The authors declare no conflicts of interest.

## Data Availability

The data that support the findings of this study are available from the corresponding author upon reasonable request.
